# Improvement of exercise and functional capacity and quality of life in patients with heart failure by iron therapy

**DOI:** 10.3389/fcvm.2023.1025957

**Published:** 2023-05-22

**Authors:** Andrea Deichl, Frank Edelmann

**Affiliations:** ^1^ Charité Universitätsmedizin Berlin, Department of Cardiology, Berlin, Germany; ^2^ DZHK (German Centre for Cardiovascular Research), Partner Site Berlin, Berlin, Germany

**Keywords:** heart faillure, iron defciency, iron therapy, exercise capacity, quality of life

## Abstract

Heart failure (HF) is one of the most common causes of death in industrialized countries and increases steadily with age. Patients with HF present many comorbidities that affect their clinical management, quality of life, and prognosis. Iron deficiency is a relevant comorbidity of all patients with heart failure. It remains the most prevalent nutritional deficiency worldwide, affecting an estimated 2 billion people and has a negative prognostic impact on hospitalization and mortality rate. To date, none of the previous studies, have provided evidence of reduced mortality or decrease in hospitalization with intravenous iron supplementation. This review describes the prevalence, clinical implications, and current trials on the treatment of iron deficiency in heart failure and discusses the Improvement of exercise and functional capacity and quality of life in patients with heart failure by iron therapy. Despite compelling evidence of the significant prevalence of ID in HF patients and current guidelines, ID is often not properly managed in clinical practice. Therefore, ID should be given greater consideration in HF health care practice to improve patient quality of life and outcome.

## Introduction

Currently, many definitions of heart failure (HF) are ambiguous and focus on different characteristics. Some approaches focus primarily on characterizing the hemodynamic and physiologic components, whereas others focus on the diagnostic elements of the clinical syndrome. A revised definition was proposed by the Japanese Heart Failure Society (JHFS), the Heart Failure Association of the European Society of Cardiology (HFA/ESC), and the Heart Failure Society of America (HFSA) in 2021. HF, like many noncategorical diseases, is regarded as a clinical syndrome since it lacks a specific pathognomonic histological or biochemical sign and could emerge from a variety of clinical disease states that are quite different from one another (1). HF is one of the most common causes of death in industrialized countries with a prevalence of 1%–2% in the adult population and increasing steadily with age (2). Numerous comorbidities are prevalent in HF patients and have an impact on their clinical care, quality of life, and prognosis. Iron deficiency (ID) represents one frequent comorbidity in patients with HF. It remains the most prevalent nutritional deficiency worldwide, affecting an estimated 2 billion people.

This review describes the prevalence, clinical implications, and current trials on the treatment of ID in HF and discusses the improvement of exercise and functional capacity, and quality of life in patients with HF by iron therapy.

## Prevalence and prognostic impact of ID in HF

ID is a relevant comorbidity in approximately 35%–50% of all patients with HF. ID is defined by current HF guidelines as a serum ferritin level of less than 100 ng/ml or when between 100 and 299 ng/ml with a transferrin saturation level of less than 20% (3). Inflammation may make it difficult to interpret blood ferritin levels. Proinflammatory cytokines can cause “iron entrapment” in macrophages, hepatocytes, and enterocytes by degrading ferroportin, the transmembrane protein responsible for iron transfer outside cells. This process often guards against pathogens that rely on iron availability for life, and it promotes a functional state of ID, rendering iron ineffective despite adequate iron storage (4).

Iron is essential for aerobic metabolism in addition to its impact on hemoglobin and myoglobin the primary proteins for O_2_ transport and accumulation. The citric acid cycle and the respiratory chain are two major pathways for oxidoreductive processes involved in the production of cellular energy. Iron sufficiency is necessary for the synthesis of mitochondrial proteins. These elements undoubtedly play a part in HF's decreased ability to exercise. Melenovsky et al. studied left ventricular samples from 91 HF patients undergoing transplantation and compared them with samples from 38 HF-free organ donors (controls). HF patients showed significantly reduced myocardial oxygen respiration and decreased activity of all tested mitochondrial enzymes as compared to controls (5). This supports the hypothesis that ID may be associated with the exacerbation of mitochondrial dysfunction already present in HF. Being highly active cells, cardiomyocytes require effective energy generation, hence, appropriate mitochondrial activity is essential. According to Bakogiannis et al. iron deficiency also hampers the electrochemical stability of cardiomyocytes due to impaired mitochondrial enzyme activity. This results in dysfunctional contractility of the cardiomyocytes and may trigger lethal arrhythmias (6). Since their production depends on the availability of iron, ferritin, and transferrin saturation blood levels in clinical settings might reveal information about iron homeostasis (4). In addition to its important role in oxygen transport and metabolism, iron also plays a critical role in microRNA biogenesis, thyroid, central nervous system, and immune system function (7).

**Figure 1:**
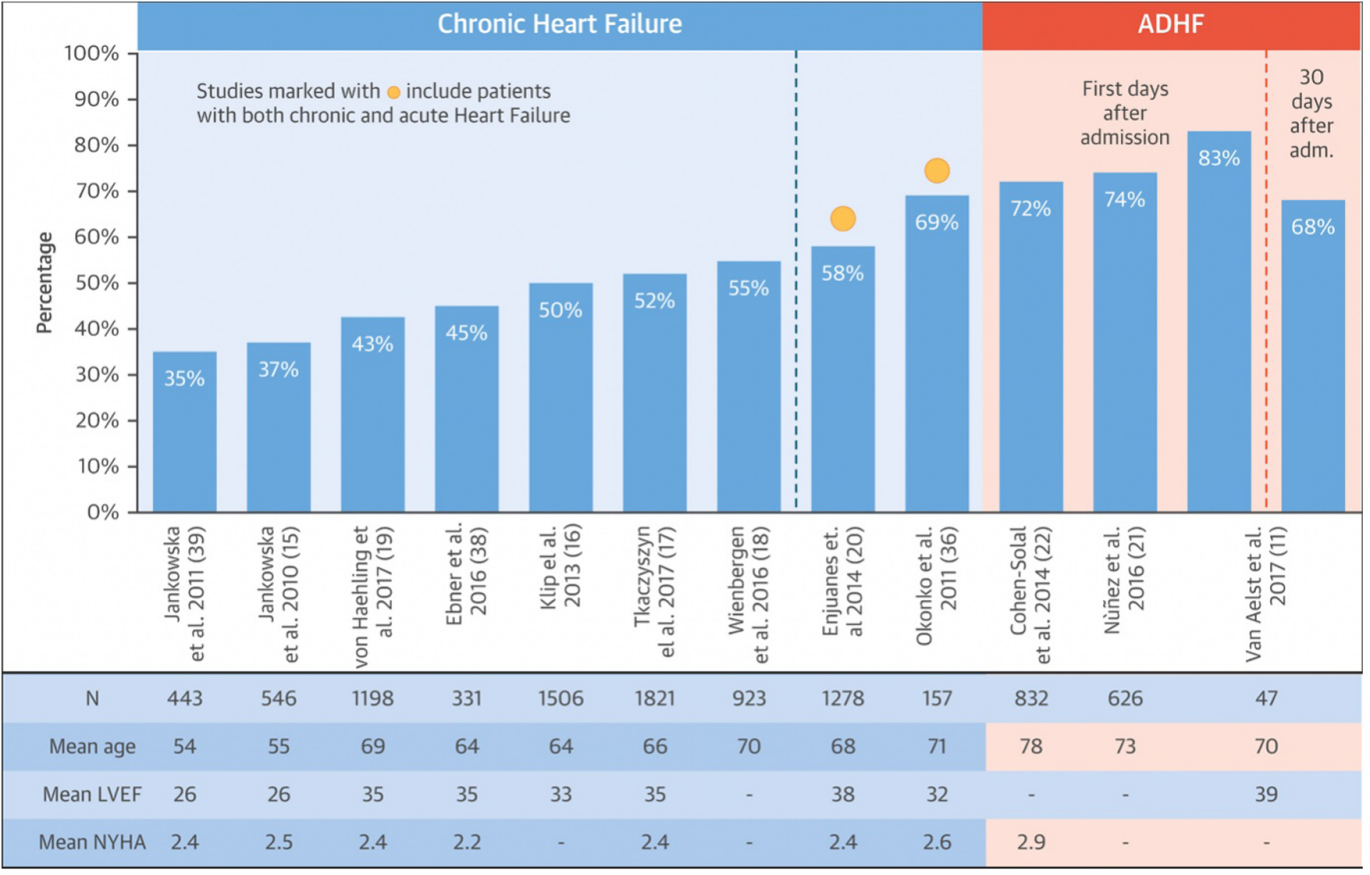
Prevalence of ID in HF. Modified after Rocha et al. (11).

Anemia in HF has a complex etiology, it is often brought on by a variety of conditions, such as hypoplastic bone marrow, insufficient erythropoiesis, gastrointestinal bleeding, drug interactions, or food-reducing absorption (8). ID persists (>30%) in individuals without anemia or abnormalities in hematologic indexes regardless of the presence of anemia (9). It can be observed that the prevalence of ID rises with the increasing NYHA class. Other risk factors such as high plasma N-terminal natriuretic peptide type B (NT-pro-BNP), high serum high-sensitivity C-reactive protein (hsCRP), and female gender also negatively affect disease progression in HF patients with ID (10). As illustrated in Figure 1, the prevalence is higher in patients with acutely decompensated HF, varying between 72% and 83% (11).

To date, none of the previous studies, nor the randomized AFFIRM-AHF trial presented at the 2020 AHA Congress, have provided evidence of reduced mortality or decrease in hospitalization with intravenous iron supplementation. However, nearly all studies were not powered for this endpoint.

Between 2016 and 2017, there were two meta-analyses devoted to IV iron therapy in patients with HF. With a total of 851 patients across 5 clinical trials, Jankowska et al. found that IV iron therapy decreased the risk of both the combined endpoint of all-cause death or cardiovascular hospitalization (OR: 0.44; 95% CI: 0.30 to 0.64; *p* < 0.0001) and the combined endpoint of cardiovascular death or hospitalization for worsening HF (OR: 0.39; 95% CI: 0.24 to 0.63; *p* = 0.0001) (12).

Using data from 4 randomized controlled trials (FER-CARS-01, FAIR-HF, EFFICACY-HF, and CONFIRM-HF) that included 839 patients with chronic HF a second metanalysis was carried out in 2017. The results showed that, compared with placebo, FCM reduced recurrent HF hospitalizations (relative risk RR: 0.41; 95% CI: 0.23 to 0.73; *p* 1⁄4 0.003) and recurrent cardiovascular hospitalizations (RR: 0.54; 95% CI: 0.36 to 0.83; *p* 1⁄4 0.004) (13).

These findings support the use of IV iron therapy in HF, which led to the creation of the prospective FAIR-HF 2 trial [NCT03036462 (14)] with a primary endpoint of the combined rate of recurrent hospitalizations for HF and cardiovascular death.

## Impact of ID on exercise and functional capacity and quality of life

Iron is an essential micronutrient involved in a wide range of biological processes, such as oxygen delivery, energy production in the mitochondria, and metabolic homeostasis.

### Exercise and functional capacity

In the published data exercise and functional capacity are frequently mentioned and used as equivalent terms but it is important to distinguish these. Contrary to functional capacity, which is described as “the ability to perform activities of daily living that require sustained, submaximal aerobic metabolism”, exercise capacity can be characterized as “the maximum amount of physical exertion that a subject can sustain” (15). Objective quantitative methods for the assessment are the 6-min walk test (6MWT—functional capacity), the graded exercise testing with electrocardiography (ECG), and cardiopulmonary exercise testing with the evaluation of peak oxygen consumption (CPET—exercise capacity).

### Quality of life—PRO

Patient-reported outcomes (PROs), as defined by the World Health Organization and the U.S. Food and Drug Administration, are patient-reported evaluations of their health status that are not interpreted by anyone else, such as physicians (16). Consequently, a patient-reported outcome measure (PROM) is an instrument, such as a questionnaire, used to quantify and gather data on a PRO, which is often associated with health-related quality of life. PROMs can be classified as general and disease-specific measures. The purpose of generic PROMs is to assess a patient's overall condition, such as her physical, functional, and emotional state, and is not limited to a specific condition. Disease-group-specific PROMs pertain to a particular set of diseases. These PROMs are often more sensitive than generic PROMs by measuring one specific disease, e.g., sleep apnea in HF patients (17). PROs are becoming more significant as predictors of mortality and hospitalization in patients with HF and can be used as primary or secondary endpoints in clinical studies, as both clinicians and patients are increasingly realizing (18). Several commonly used questionnaires have been validated in HF for assessing the quality of life, including the Kansas City Cardiomyopathy Questionnaire (KCCQ), the Minnesota Living with Heart Failure Questionnaire (MLHFQ), the European Quality of Life 5 Dimensions (EQ-5D) or the Short Form 36 (SF-36) (19, 20). In a recently published cohort study of 2021, 2,872 patients with chronic HF with reduced ejection fraction have been enrolled with an evaluation of NYHA class and KCCQ-OS data at baseline and 12 months. Examined were the longitudinal changes and correlations between the two measures, NYHA and KCCQ-OS, from baseline to 12 months, with clinical outcomes occurring between months 12 and 24. The cohort analysis demonstrated that the patient-reported KCCQ-OS was more likely than the clinician-assigned NYHA class to detect a substantial change in health status over time (21).

Evaluating PROs for sex and gender differences reveals consistent evidence that women and men perceive their physical symptoms and psychological load differently. Most investigations discovered gender discrepancies in PRO, with female patients faring worse than male patients (22, 23). In the process of developing and validating PROMs, it is critical to have a gender-balanced population, examine discrepancies between male and female reports, and consider gender identities beyond the binary idea. However, despite the importance of these PROs, the chance to incorporate them into standard clinical practice is seldom taken advantage of. The practicality of integrating PROMs into clinical workflow, linking it to medical records, interpretability of the data, and the expense of collecting and analyzing the data are just some of the hurdles to the regular use of PRO (24). The most prevalent formal HF-specific PROMs were designed primarily for outpatient usage and were not originally intended for hospitalizations (25). Nonetheless, these PROMs may have therapeutic applications in the inpatient context and, if utilized effectively, may enhance risk classification and patient-centered treatment.

## Clinical trials—oral iron therapy

Two clinical studies the IRON-5 trial (Short Term Oral Iron Supplementation in Systolic Heart Failure Patients Suffering From Iron Deficiency Anemia) and the IRONOUT HF trial (Oral Iron Repletion Effects On Oxygen Uptake in Heart Failure) assessed the effects of oral iron supplementation in comparison to intravenous iron (IV) supplementation in patients with HF (summarized in Table 1).

**Table 1:** Main trials of iron supplementation in heart failure. Modified after Rizzo et al. (4).

Trial	Pts (n)	Diagnosis	Design	Dosage	Inclusion criteria	Primary endpoint
FERRIC HF 2008	35	HFrEF	2:1 (Iron:placebo)	Iron sucrose 200 mg weekly until ferritin >500 ng/ml	CHF (NYHA II or III) with LVEF ≤ 45%	Change in absolute pVO2 from baseline to week 18
FAIR-HF 2009	459	HFrEF	2:1 (FCM:placebo)	200 mg ferric carboxymaltose	CHF (NYHA II or III), LVEF of 40% or less (for patients with NYHA II) or 45% or less (for NYHA III)	Change in PGA and NYHA class from baseline to week 24
IRON-5 HF 2013	54	HF	1:1 (iron:placebo)	Ferrous Sulfate 200 mg 3 times daily for 90 days	LVEF < 50% NYHA II-III (able to perform 6MWT)	Change in 6MWT from baseline to 90 days
CONFIRM-HF 2014	304	HFrEF	1:1 (FCM:placebo)	200 mg ferric carboxymaltose	Ambulatory symptomatic HF patients with LVEF ≤ 45%	Change in 6 MWT distance from baseline to Week 24
IRONOUT 2016	225	HFrEF	1:1 (FCM:placebo)	Oral Iron 150 mg Polysaccharide iron complex (Feramax) twice daily	LVEF ≤ 40% with NYHA II through IV symptoms	Change in peak VO2 from baseline to 16 weeks
EFFECT-HF 2017	174	HFrEF	1:1 (FCM:standard of care)	500 mg ferric carboxymaltose 3 times	HFrEF (LVEF ≤ 45%)	Change in peak VO2 from baseline to 24 weeks measured by CPET
AFFIRM-AHF 2020	1132	AHF after restabilization	1:1 (FCM:placebo)	500–1,500 mg ferric carboxymaltose according to Hb and weight value	Patients Admitted for Acute Heart Failure with LVEF ≤ 50%	The composite of recurrent HF hospitalizations and CV death up to 52 weeks

The IRON 5 trial, published in 2013, randomized patients with a LVEF of <50% and NYHA class II to IV, a hemoglobin concentration between 9 and 12 g/dl, a transferrin saturation (TSAT) level of >20%, and a ferritin concentration of <500 µg/dl. Patients were randomly allocated to three groups with three distinct treatments using a double-blind method: 1. oral ferrous sulfate treatment, 200 mg three times a day, plus IV placebo, 2. oral placebo plus IV iron sucrose, 200 mg weekly, and 3. oral and IV placebo. Patients received oral iron for 8 weeks, whereas IV iron was administered over 5 weeks. The primary endpoint was the change in peak oxygen consumption (peak VO_2_) measured by ergospirometry throughout a 3-month follow-up period. The trial was stopped early with only 23 randomized subjects due to financing issues. After 3 months, the IV iron group's peak oxygen consumption significantly increased, whereas the other groups did not (26).

A phase II double-blind, randomized, placebo-controlled trial called IRONOUT was also published in 2017. It included 225 patients with HF, an LVEF of <40%, and ID, which was defined as a ferritin concentration of 15–100 μg/L or ferritin 101–299 μg/L with TSAT of <20%. Patients were given 150 mg of iron polysaccharide twice a day for 16 weeks. The primary endpoint was the difference in peak oxygen uptake from baseline to 16 weeks, which did not vary significantly between the two groups at the end of the study (27).

Several clinical trials, such as those mentioned above, have had inconclusive outcomes for oral administration, therefore oral iron supplementation remains contentious. In addition, it is important to note that up to 40% of patients report side effects, most often gastrointestinal discomfort such as nausea, flatulence, stomach pain, diarrhea, and constipation (10). The ESC guidelines thus suggest IV iron supplementation for HF patients with ID as oral treatment does not adequately replenish iron reserves (28).

## Clinical trials—intravenous iron therapy

As summarized in Table 1, several major studies have investigated intravenous iron supplementation and shown significant benefits in improved exercise and functional capacity. The first large-scale trial, FAIR-HF (Ferinject Assessment in patients with IRon deficiency and chronic Heart Failure) enrolled 459 patients with NYHA class II (LVEF ≤ 40%) or NYHA class III (LVEF ≤ 45%) and a hemoglobin concentration ranging between 9.5 and 13.5 g/dl. ID was defined as a serum ferritin concentration of <100 µg/L or a ferritin concentration range from 100 to 300 µg/L with a TSAT of <20%. Patients were randomly assigned to receive either IV ferric carboxymaltose (FCM) or a placebo 2:1. FCM was administered weekly during the correction phase and then every 4 weeks during the maintenance phase until iron repletion was accomplished. The trial demonstrated that IV FCM improved the main outcome, the self-reported patient global assessment (PGA), which compares the patient's present well-being to their prior assessment. Secondary endpoints included NYHA functional class, 6-min walk distance, and quality of life as measured by the Kansas City Cardiomyopathy Questionnaire, all of which showed a statistically significant improvement regardless of the presence of anemia at baseline (47% had an NYHA functional class I or II at week 24 after IV FCM, as compared with 30% of patients assigned to placebo; 6MWT increased from 274 ± 6 to 313 ± 7 m after 24 weeks; mean KCCQ increased from 52 ± 1 to 66 ± 1 points) (29). The CONFIRM HF trial validated these findings (A Study to Compare the Use of Ferric Carboxymaltose With Placebo in Patients With Chronic Heart Failure and Iron Deficiency). This mentioned trial involved 304 patients with symptomatic HF, NYHA class II or III, a LVEF of ≤45%, and an elevated level of either NT-proBNP or B-type natriuretic peptide. Patients were given a 1:1 ratio of either a placebo or an iron dose ranging from 500 to 2,000 mg during the first 6 weeks of the trial. If ferritin and/or TSAT remained to exhibit ID beyond this period, participants received 500 mg of FCM at each visit in weeks 12, 24, and 36. The main outcome was the change in the 6MWT from baseline to 24 weeks, which was significantly improved in the IV FCM group compared to the placebo group (difference: 33 ± 11 m; *p* = 0.002). Quality-of-life scores were significantly improved in FCM participants compared with placebo-treated patients on the KCCQ and the EQ-5D questionnaire (30).

In the 2017 EFFECT-HF study (Effect of Ferric Carboxymaltose on Exercise Capacity in Patients with Chronic Heart Failure and Iron Deficiency), 174 patients with LVEF ≤ 45% and predominantly NYHA class II at baseline were randomized, in a nonblinded fashion, to IV FCM therapy or standard of care for a 24-week follow-up. The main outcome was the change in peak VO_2_ between baseline and 24 weeks, as measured by CPET. After 24 weeks, peak oxygen decreased in the control group (−0.16 ± 0.387 ml/min/kg; *p* = 0.02) (31).

Positive effects for HF hospitalization rates and reduction in cardiovascular mortality rates can be inferred from the results of data from meta-analyses but have yet to be confirmed in a prospective study (12).

The randomized, double-blind, placebo-controlled AFFIRM-AHF study assesses the influence of intravenous FCM on hospitalizations and mortality in iron-deficient patients treated for acute HF. Patients hospitalized for acute HF with an LVEF of <50% and NYHA class II or III were recruited for the trial. Patients were randomly assigned to receive either IV FCM as an active treatment or a placebo. To qualify, patients' blood ferritin levels are required to be <100 or 100–299 µg/L with a TSAT of <20%, according to the ID criteria used in the previous trial. The trial's main outcome was a composite of HF hospitalizations and cardiovascular deaths over a 1-year follow-up period. At 52 weeks of follow-up, the incidence of cardiovascular death did not vary between the treatment and placebo groups. For the combined primary endpoint of total hospitalizations and cardiovascular (CV) death, the total number of events was numerically reduced in those treated with IV FCM compared to placebo by 21%, although this difference was not statistically significant (RR 0.79; 95% CI 0.62–1.01) (32).

As shown in Table 1 the most researched IV formulations for HF are IS and FCM. According to the labeling for the individual products, the dosage regimens for FCM and IS are different. While IS dosage is limited to 200/300 mg iron per infusion and 600 mg once weekly, FCM can be provided in quantities of up to 1,000 mg iron in a single infusion. From an economic perspective, the main disadvantage of IS is the limited dosage per session that requires multiple sessions, even if IS is cheaper than FCM. Nevertheless, economic analyses support the cost-effectiveness of FCM, which is driven by improved symptomatic status (NYHA class) and a drop in hospitalization rates (33). Because a complete iron dose may be delivered in fewer infusions with FCM compared to other formulations, the former may have cheaper expenses per therapy (34).

## Ongoing trials

There are now several clinical trials being undertaken, the majority of which concentrate on the use of IV iron therapy in patients with chronic HF employing strict primary endpoints such as CV death or HF hospitalization.

◾ **FAIR-HFpEF** (Effect of IV Iron in Patients With Heart Failure With Preserved Ejection Fraction; NCT03074591)

The FAIR-HFpEF trial examines whether treatment with IV FCM for patients with heart failure with preserved ejection fraction (HFpEF) and ID, both with or without anemia, can increase exercise capacity as determined by a 6-min walking test (primary endpoint after 52 weeks). Secondary endpoints are the PGA quality of life questionnaire, the difference in NYHA class, and the impact on mortality and HF-related hospitalization rates from baseline to the conclusion of the trial (35).

◾ **HEART-FID** (Randomized Placebo-controlled Trial of FCM as Treatment for Heart Failure With Iron Deficiency; NCT03037931)

The HEART-FID trial is a double-blind, multicenter, prospective, randomized, placebo-controlled trial aimed to evaluate the efficacy of intravenous FCM over that of a placebo. The combined primary endpoint is a composite of death and HF hospitalization at 12 months and changes from baseline to 6 months in the 6MWT distance (36).

◾ **FAIR-HF2** (Intravenous Iron in Patients With Systolic Heart Failure and Iron Deficiency to Improve Morbidity & Mortality; NCT03036462)

The clinical trial is designed as an international, prospective, multicenter, double-blind, parallel-group, randomized, controlled, interventional trial to determine whether long-term therapy with IV FCM compared to placebo can reduce the rate of recurrent HF hospitalizations and CV death in patients with heart failure with reduced ejection fraction (HFrEF). The major outcome measure is the combined rate of recurrent hospitalizations for HF and cardiovascular death (number of events) (14).

◾ **IRONMAN** (Intravenous Iron Treatment in Patients With Heart Failure and Iron Deficiency; NCT02642562)

Compared to other trials investigating iron therapy, IRONMAN will use a different type of iron therapy, IV iron isomaltose (IIM), compared to FCM used in the above-mentioned trial.

IIM is compared to FCM dextran-based. They differ in type, structure, half-life, stability of polymerization, pharmacokinetics, dosage, and tolerability (37). The European Medicines Agency (EMA) issued a positive risk-benefit assessment for both iron preparations. However, the current evidence for the clinical benefit of i.v. iron supplementation primarily relates to FCM, such that FCM is also the only i.v. iron supplementation is recommended in the ESC guidelines for the treatment of HF patients with ID.

The IRONMAN trial is a randomized, open-label multicentre trial compared to the other trials. The purpose of the trial was to determine whether the treatment of IV IIM in addition to standard treatment would enhance the prognosis for patients with HF and ID. The primary endpoint is CV mortality or hospitalization for worsening HF (14).

◾ **RESAFE-HF trial** (Iron Intravenous Therapy in Reducing the burden of Severe Arrhythmias in HFrEF; NCT04974021)

The RESAFE-HF trial is a prospective, single-center, and open-label registry trial, to evaluate the effect of IV FCM on HFrEF patients' iron stores, arrhythmic burden, hospitalizations, functional capacity, quality of life, ultrasonographic parameters, and disease biomarkers. Patients with HFrEF and CIEDs scheduled to receive IV FCM as a treatment for ID were eligible to participate. The primary endpoint is a composite endpoint of haemoglobin ≥12 g/dl, ferritin ≥50 ng/L, and transferrin saturation >20%. Secondary endpoints include unplanned HF-related hospitalizations, ventricular tachyarrhythmias detected by CIEDs and Holter monitors, echocardiographic markers, functional status (VO_2_ max and 6 min walk test), blood biomarkers, and quality of life (38).

## ESC guidelines

The updated 2021 European Society of Cardiology (ESC) guidelines for the diagnosis and management of acute and chronic HF emphasize the importance of ID diagnostics. It is now a class Ic recommendation that all patients with HF be screened periodically for anemia and ID, with a full blood count, serum ferritin concentration, and transferrin saturation. Regarding discharge of patients with HFrEF, recently hospitalized for worsening HF, intravenous FCM supplementation should also be considered to reduce HF rehospitalizations and symptom burden (28).

**Figure 2:**
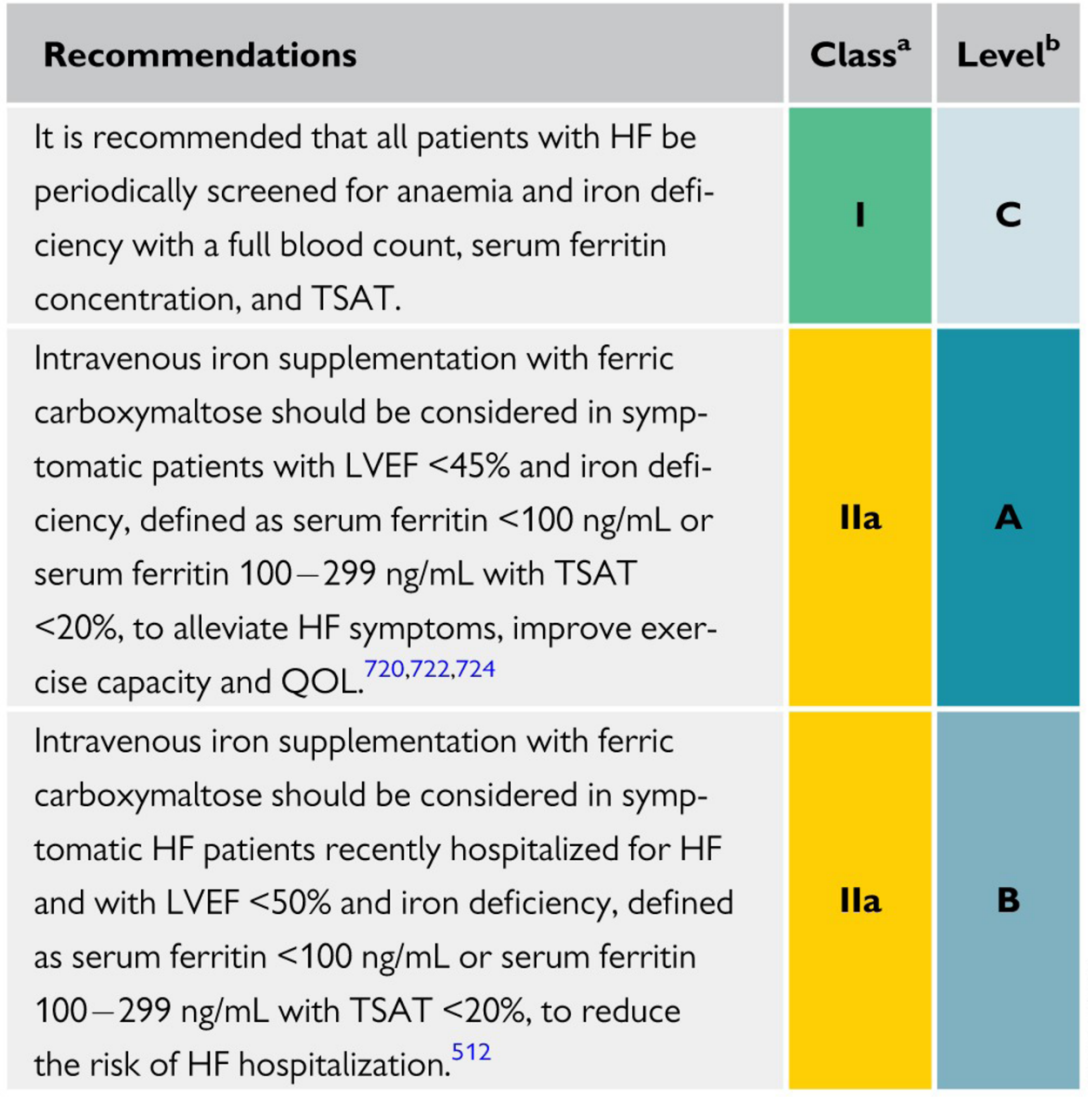
Recommendations for the management of anemia and iron deficiency in patients with heart failure (28).

There is no update to the 2016 published guidelines regarding intravenous iron supplementation. It remains a class IIa recommendation that patients with ID and symptomatic HFrEF receive intravenous FCM to improve exercise and functional capacity and quality of life. Iron therapy is propagated for symptomatic patients with HF when ferritin is <100 μg/L or when ferritin is between 100 and 299 μg/L and transferrin saturation is <20% (39) (Figure 2).

## Conclusion

Iron deficiency is a relevant co-morbidity in HF patients, which has a poor prognostic impact on hospitalization and mortality rates while impairing physical and functional performance and quality of life. Despite compelling evidence of the significant prevalence of ID in HF patients and current guidelines, ID is often not properly managed in clinical practice. Therefore, ID should be given greater consideration in HF health care practice to improve patient quality of life and outcome. Furthermore, numerous issues are still unsolved despite increased research efforts to understand the function of ID in HF. The mechanistic research now being done to explain the advantages of IV iron is cardio-centric in character, even though iron's functions are not only related to the cardiovascular system. Understanding the pathophysiology of ID will facilitate research into the effects of IV iron on the entire circulation.

## Data Availability

The original contributions presented in the study are included in the article, further inquiries can be directed to the corresponding author.
